# Chemical Weathering and CO_2_ Consumption Inferred from Riverine Water Chemistry in the Xi River Drainage, South China

**DOI:** 10.3390/ijerph20021516

**Published:** 2023-01-13

**Authors:** Yanpu Zhao, Jan R. Wijbrans, Hua Wang, Pieter Z. Vroon, Jianghao Ma, Yanqiong Zhao

**Affiliations:** 1 Key Laboratory of Tectonics and Petroleum Resources, Ministry of Education, China University of Geosciences, Wuhan 430074, China; 2 School of Earth Resources, China University of Geosciences, Wuhan 430074, China; 3 Department of Earth Sciences, Vrije Universiteit Amsterdam, 1081 HV Amsterdam, The Netherlands; 4 School of Economics and Management, China University of Geosciences, Wuhan 430074, China

**Keywords:** chemical weathering, CO_2_, sulfuric acid, Xi River drainage

## Abstract

Hydrochemistry and strontium isotope data were analysed in water samples from the Xi River Drainage system to reveal the spatial and seasonal variations in chemical weathering, associated CO_2_ consumption fluxes, and their control factors. The main ions were Ca^2+^, Mg^2+^, and HCO_3_^−^, which are characteristic of a drainage system on carbonate-dominated bedrock. The dissolved loads were derived from four major end-member reservoirs: silicate, limestone, dolomite, and atmosphere. The silicate weathering rates (SWRs) increased downstream from 0.03 t/km^2^/year to 2.37 t/km^2^/year. The carbonate weathering rates (CWRs) increased from 2.14 t/km^2^/year in the upper reaches, to 32.65 t/km^2^/year in the middle reaches, and then decreased to 23.20 t/km^2^/year in the lower reaches. The SWR values were 281.38 and 113.65 kg/km^2^/month during the high- and low-water periods, respectively. The CWR values were 2456.72 and 1409.32 kg/km^2^/month, respectively. The limestone weathering rates were 2042.74 and 1222.38 kg/km^2^/month, respectively. The dolomite weathering rates were 413.98 and 186.94 kg/km^2^/month, respectively. Spatial and seasonal variations in chemical weathering were controlled mainly by lithology, vegetation, and climate (temperature, water discharge, and precipitation). The CO_2_ consumption flux by chemical weathering was estimated at 189.79 × 10^9^ mol/year, with 156.37 × 10^9^ and 33.42 × 10^9^ mol/year for carbonate and silicate weathering, respectively. The CO_2_ fluxes by chemical weathering are substantially influenced by sulfuric acid in the system. The CO_2_ flux produced by sulfuric acid weathering was estimated at 30.00 × 10^9^ mol/year in the basin. Therefore, the Xi River Basin is a CO_2_ sink with a net consumption of CO_2_ flux of 3.42 × 10^9^ mol/year.

## 1. Introduction

Chemical weathering plays an important role in surface processes that link the rock cycle of the solid earth to the hydrological cycle of rivers, oceans, and the atmosphere. Rock weathering under the influence of dissolved carbonic acid consumes atmospheric CO_2_ and produces HCO_3_^−^ and CO_3_^2−^, which are eventually discharged into the sea where they are absorbed in the marine carbonate buffer system. The global carbon cycle, in concert with topography development on the continents, plays an important role in regulating global climate, as topography provides a fresh weatherable surface and enhances the consumption of the greenhouse gas CO_2_.

The chemical weathering of terrestrial silicate rocks constitutes a significant carbon sink in global biogeochemical cycles on geological time scales [1,2,3,4,5,6]. The CO_2_ consumption flux by silicate rock weathering controls the long-term global carbon cycle time scales of millions of years [7,8,9,10]. This consumed CO_2_ is chemically locked in marine sediments and cannot be easily released back into the atmosphere on short timescales. Silicate rock weathering, along with organic carbon burial, thus controls the atmospheric CO_2_ content on million- to 100-million-year geological time scales and drives the evolution of the global climate [10,11]. The chemical weathering of carbonate, perhaps surprisingly, also consumes atmospheric CO_2_. This cycle releases CO_2_ back into the atmosphere through carbonate deposition. The chemical weathering of continental carbonates and deposition in the ocean establishes a closed carbon cycle, which requires a relatively short time for its balance when compared to the silicate weathering control cycle. Carbonate dissolution consumes approximately 12.3 × 10^12^ mol of carbon per year [11]. The inorganic carbon fluxes from carbonate rock weathering are carbon sinks on the 10^2^- to 10^3^-year geological timescale [10,12]. For further research into the carbon sink associated with chemical weathering and their controlling mechanisms, the contribution of each end-member must be more accurately determined, especially in rivers as they represent short circuits between the terrestrial and marine water cycles.

During chemical weathering, sulfuric acid as a strong acid formed by atmospheric SO_2_ causes the weathering of carbonate rocks, leading to CO_2_ production rather than CO_2_ sequestration because the retention time of SO_4_^2−^ (10^7^ years) is longer than that of HCO_3_^−^ (10^5^ to 10^6^ years) in the ocean. Fundamentally, sulfuric acid as a strong acid dominates both carbonate and the silicate weathering cycles at the expense of carbonic acid as a weak acid [1,2,13,14]. When carbonate minerals precipitate, half of HCO_3_^−^ reacts to form gaseous CO_2_ in the atmosphere and aqueous CO_2_ in the ocean. Unless gypsum or barite can precipitate, the participation of sulfuric acid in carbonate weathering is essentially a net release of CO_2_ into the atmosphere [10,15]. If the sulfuric acid weathering of carbonate rocks is ignored, the CO_2_ consumption involved in silicate weathering will be overestimated. Sulfuric acid is mainly derived by the precipitation of sulfides through the oxidation of natural sulfides and of human activities. South China is one of the world’s three largest regions with respect to anthropogenic acid rain deposition due to SO_2_ emissions related to coal combustion [16,17]. Global atmospheric carbon dioxide consumption due to chemical weathering is reduced by approximately 13% due to sulfuric acid weathering [2,14,18]. Therefore, ascertaining the role of H_2_SO_4_ in chemical weathering is essential to accurately estimate the CO_2_ consumption flux in the Xi River drainage.

The Xi River Is China’s third-longest river, with part of its drainage system in Guangdong province, which is severely affected by anthropogenic acid rain. Carbonate rocks are widely exposed in the Xi River drainage basin, which makes it especially important for analysing the interplay of natural and anthropogenic chemical weathering processes. Previous studies have discussed the hydrochemistry and chemical weathering processes of the basin at different temporal and spatial scales [2,10,19,20]. These studies focused on the effects of rock lithology, water temperature, exogenous acid, and other factors related to the weathering process, whereas they did not adequately analyse the contributions of the carbonate rock end-members (limestone and dolomite). In this study, we systematically investigated the chemistry and strontium isotopic data of Xi River water. This research was aimed at revealing the spatial and seasonal variations in chemical weathering, associated CO_2_ budgets, and their control factors. Another purpose was to discuss the effects of H_2_SO_4_ on the weathering process in a typical karst region affected by acid rain in South China.

## 2. Study Area

### 2.1. Geography

**Figure 1:**
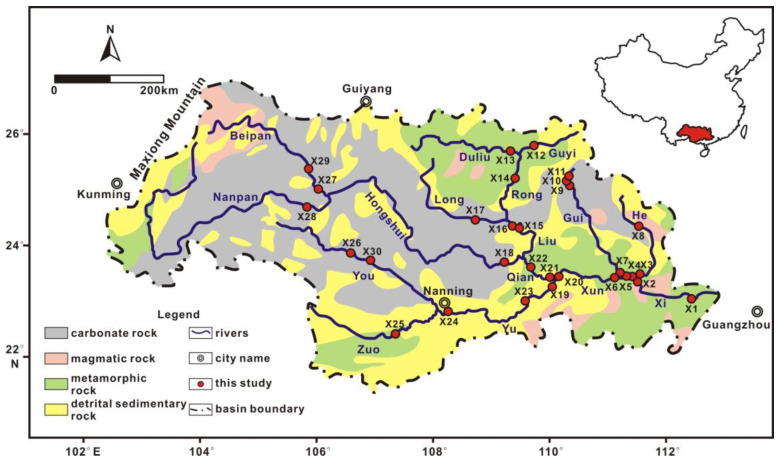
Geological map of the Xi River Basin and sampling locations [21].

The Pearl River drainage basin is located in South China, ranging from 21° to 27° N latitude and from 102° to 114° E longitude. As the principal trunk stream of the Pearl River, Xi River drainage accounts for 77.8% of the Pearl River drainage area and provides 63.9% of its water discharge. The drainage area of the Xi River is 353,100 km^2^, with a length of 2075 km [10]. It originates from Maxiong Mountain, runs through southern China, and eventually flows via the Pearl River delta into the South China Sea near the towns of Guangzhou, Hong Kong, and Shenzhen (Figure 1). The Xi River flows through Yunnan, Guizhou, Guangxi, and Guangdong Provinces. The drainage system consists of five main tributaries, including the Beipan, Liu, Gui, He, and Yu Rivers. The topography of the Xi River Basin consists of the Yunnan–Guizhou Plateau, Guangxi Basin, and Pearl River Delta from west to east. Ninety-four percent of the Xi River drainage area is covered with mountainous or hilly country.

### 2.2. Geology

The rocks exposed in the Xi River Basin date from the Precambrian to Quaternary era (Figure 1). Karstified limestone landscapes are widely distributed in the upper reaches; Permian to Triassic carbonate rocks cover an area of 155,000 km^2^, accounting for 44% of the total Xi River drainage basin. Coal deposits interbedded with carbonates are rich in sulfides. Precambrian metamorphic rocks and magmatic rocks can be found in the lower reaches. The dominant magmatic rocks are associated with the Mesozoic Yanshanian granite suite and include intermediate-acidic intrusive and extrusive rocks. A smaller component of magmatic rocks is that they are Permo-Triassic and early Paleozoic in age. Jurassic clastic sedimentary rocks are scattered in the middle reaches of the basin.

### 2.3. Climate and Human Activities

The Xi River Basin has a humid subtropical climate with an average yearly temperature of 14–22 °C [10,22]. The mean annual precipitation is 1470 mm. The mean annual evaporation varies from 900 to 1600 mm [10].

By the end of 2020, the Xi River Basin was home to approximately 66.5 million people. There are rich agricultural lands and mineral resources in the upper reaches. The agricultural area, including dry land and paddy fields, is approximately 40,000 km^2^ in Guangxi Province. Crops are grown in dry land with various types of fertilizers and/or pesticides. Paddy fields are favorable for rice with relatively simple fertilizers and/or pesticides. The Hechi region in the upper course is an important nonferrous metal mining area in South China. Arsenic reserves in the Nandan region account for 19% of global reserves. There is an important lead-zinc mining area in Wuxuan located in the middle reaches. Most mineral resources in the Xi River Basin are currently being mined, although some resources are in the post-mining and mine remediation stages.

## 3. Sampling and Analysis Methods

The high-water period of Xi River drainage lasts from April to September, whereas the low-water period lasts from October until March. Water samples covering 30 sites were collected in July and October 2019. The samples were collected in the main stream, first-level, and second-level tributaries of the Xi River drainage (Figure 1).

Water samples were collected at a depth of 0.3 m. The samples were filtered through 0.45 μm cellulose acetate lipid membranes and then stored in polyethylene bottles rinsed 3–4 times with water. The water samples were acidified with ultrapure nitric acid to a pH < 2 to prevent algal growth. All samples were stored and refrigerated at 4 °C for further analysis.

The major ion concentrations (K^+^, Na^+^, Ca^2+^, Mg^2+^, Cl^−^, SO_4_^2−^, NO_3_^−^) were determined via an ion chromatograph (IC 925) with an analysis error of less than 5%. The accuracy of the cation and anion concentrations was 0.001 mg/L. The concentrations of HCO_3_^−^ were determined via an ultraviolet spectrophotometer. The concentrations of SiO_2_ were measured using the silicomolybdic yellow colorimetric method with an accuracy of 0.01 mg/L. Ion analyses were carried out at China University of Geosciences. Strontium was purified by extraction chromatography. Then, strontium was converted to nitrate and dissolved in a nitrous solution for measurement. The strontium isotope compositions were measured via a Thermofisher Neptune MC-ICPMS at Vrije University Amsterdam.

## 4. Results

### 4.1. Major Ions

Table 1 shows the basic parameters, major ions, and Sr isotope data in Xi River water. The Xi River waters had pH values of 7.45 to 8.51 with an average of 8.00 during the high- water period, whereas the pH values ranged from 7.36 to 8.48 with a mean of 7.87 during the low-water period, which is consistent with water in contact with limestone and dolomite. The water samples were slightly alkaline. The high pH values reflect the importance of the dissolution of carbonate in the drainage. The water coming from typical carbonate sediment has a high pH value, which is fairly constant at approximately 8.3, as is to be expected for water in equilibrium with dissolved CaCO_3_ and atmospheric CO_2_. Lower values appeared in rivers exposed to silicate rock where the carbonate buffering is less effective. Water samples in equilibrium with atmospheric CO_2_ and no carbonate buffering have pH’s of ca 5.3. Areas with anthropogenic atmospheric SO_2_ and limited carbonate buffering also have lower pH values and, in the absence of carbonate buffering, much lower values.

**Figure 2:**
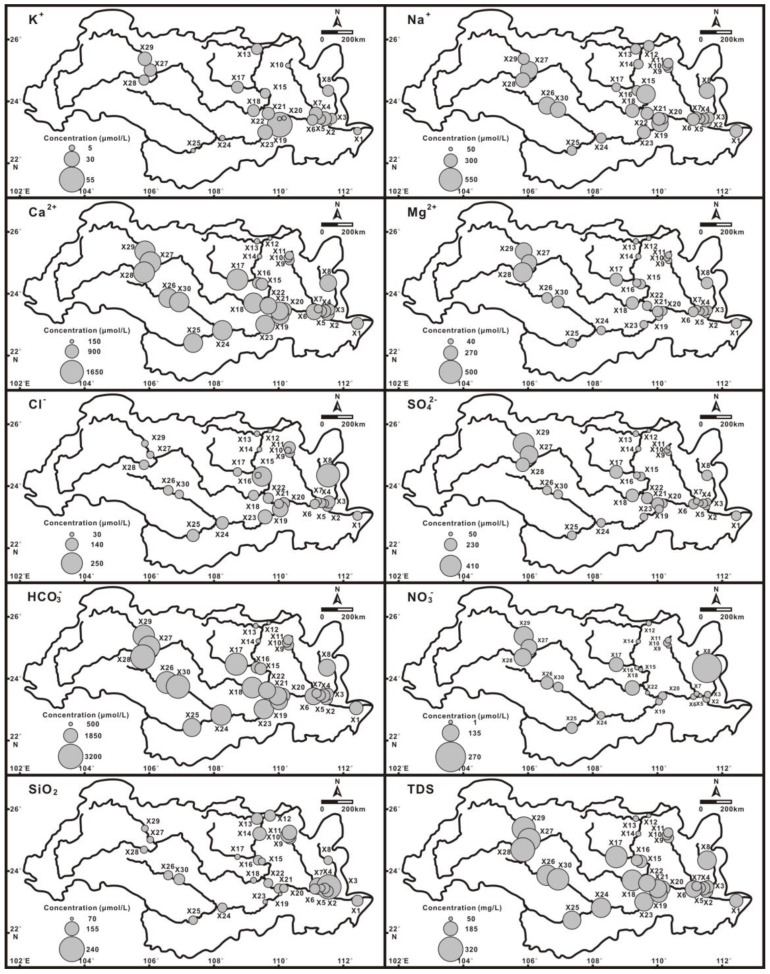
Spatial distributions of weighted averages of major ions, dissolved silica, and total dissolved solids (TDS) in the Xi River Basin.

The concentrations of total dissolved solids (TDS) widely varied from 49.84 to 305.61 mg/L during the high-water period (average: 193.22 mg/L) and 82.7~330.5 mg/L during the low-water period (average: 204.5 mg/L), similar to the Mackenzie River, Yalong River, and Han River draining carbonate-dominated regions [23,24,25,26,27]. Seasonal variation is related to the dilution effect of water discharge. However, the ratio of the dilution and the increase in water discharge was not 1. During the low-water period, a portion of the exposed surface in the basin may be out of contact with river water and therefore be unavailable for water–rock interaction. In contrast, during the high-water period, more surface area is in contact with river water, and chemical weathering is enhanced [8,28,29]. The TDS concentrations decreased downstream along the main trunk (Figure 2). Total cation concentrations (TZ^+^ = K^+^ + Na^+^ + 2Ca^2+^ + 2Mg^2+^) ranged from 571 to 4015 μeq/L and from 801 to 4437 μeq/L during the high- and low-water periods, which is within the range of variation of the world’s 61 largest rivers [11]. The total anion concentrations (TZ^−^ = HCO_3_^−^ + 2SO_4_^2−^ + NO_3_^−^ + Cl^−^) ranged from 543 to 3822 μeq/L and from 1018 to 4164 μeq/L during the high- and low-water periods, respectively. The TZ^+^ of most samples was slightly higher than the TZ^−^ for the normalized ionic charge balance values [NICB = (TZ^+^ − TZ^−^)/(TZ^+^ + TZ^−^) × 100%] within ±5%. The slight imbalance may be attributed to unanalysed organic complex matter [8,30].

**Figure 3:**
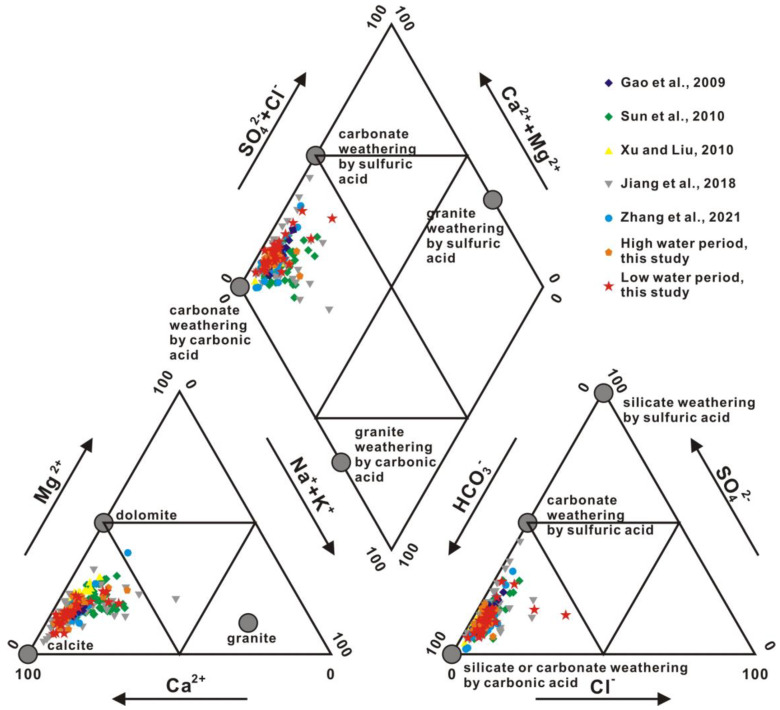
Piper diagram of the Xi River Basin with end-member compositions for carbonic and sulfuric acid weathering of major rock types [2,10,20,22,28].

Figure 2 and Figure 3 show the characteristics of the major ion compositions. The predominant cation in Xi River water was Ca^2+^, which composed over 50% of the total cations, followed by Mg^2+^, and then Na^+^ + K^+^. The Ca^2+^ concentrations were 157~1580 μmol/L and 254~1703 μmol/L during high- and low-water periods, respectively. The Mg^2+^ concentrations were 70~420 and 38~495 μmol/L, respectively. HCO_3_^−^ was the main anion, whose concentrations ranged from 408 to 3095 μmol/L and from 764 to 3250 μmol/L during high- and low-water periods, respectively. The second major anion was SO_4_^2−^ (46~346 and 55~536 μmol/L, respectively). The Cl^−^ concentrations were 31~181 μmol/L and 39~799 μmol/L, respectively. The NO_3_^−^ concentrations were 0~202 and 0~445 μmol/L. Most of the major ions show distinct seasonal variations, with low contents during the high-water period and high contents during the low-water period. Ca^2+^, Mg^2+^, and HCO_3_^−^ predominantly originate from chemical weathering [30,31]. The concentrations of Ca^2+^, Mg^2+^, and HCO_3_^−^ decrease downstream along the trunk (Figure 2). Spatial variations in ion concentrations are caused by lithologic distribution. The wide carbonate terrain, especially the karst topography in the upper course, provides advantageous conditions for carbonate chemical weathering. The lower course inherits dissolved loads from the upper course when flowing through the silicate terrain. The silicate weathering rate is much slower than that of carbonate under the same conditions, indicating that Ca^2+^, Mg^2+^, and HCO_3_^−^ transported in the upper reaches are much more abundant than Na^+^ + K^+^ originating from silicate weathering [30,32]. Thus, all samples were of the HCO_3_^—^Ca/Mg type, showing that the water chemistry was dominated by carbonate weathering.

### 4.2. Strontium Isotopes

**Figure 4:**
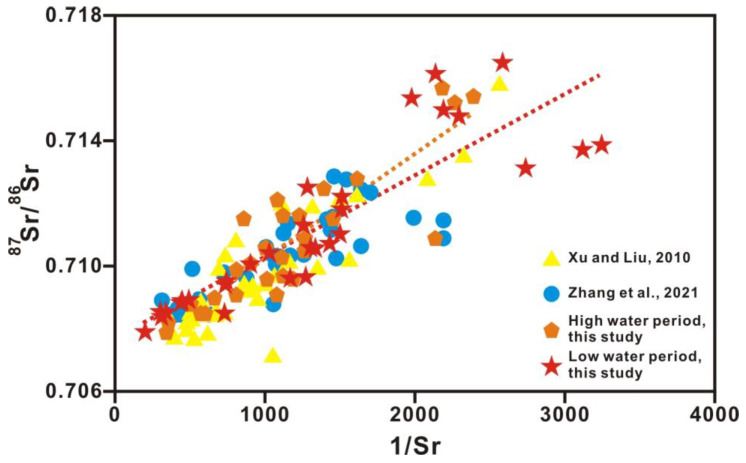
The relationship between ^87^Sr/^86^Sr and 1/Sr in dissolved water samples in the Xi River Basin. The orange dashed line indicates the trend line between 1/Sr and ^87^Sr/^86^Sr during the high-water period. The red dashed line indicates the trend line between 1/Sr and ^87^Sr/^86^Sr during the low-water period. Refs. (Xu and Liu, 2010; Zhang et al., 2021) are cited in figure [10,22].

Strontium concentrations in the Xi River drainage ranged from 0.418 to 2.915 μmol/L in the high-water period (average: 1.090 μmol/L) and from 0.308 to 4.975 μmol/L in the low-water period (average: 1.260 μmol/L), higher than the average of 0.89 μmol/L calculated for the world rivers [33]. The spatial variation in strontium concentrations in the dissolved load of the Xi River drainage followed that of Ca^2+^, Mg^2+^, and HCO_3_^−^. The ^87^Sr/^86^Sr ratios in the Xi River drainage varied from 0.7079 to 0.7157 in the high-water period (average: 0.7108) and from 0.7079 to 0.7165 in the low-water period (average: 0.7112), which is lower than the global average value of rivers (0.7119). The ^87^Sr/^86^Sr ratios increased downstream along the main trunk. A quarter of the samples had ^87^Sr/^86^Sr ratios ranging from 0.707 to 0.709, corresponding to Phanerozoic marine carbonate values (0.7065~0.709) [34]. The variable strontium concentrations and ^87^Sr/^86^Sr ratios clearly reflect the different types of exposed rocks in the Xi River Basin. The upper reaches that drain a region of carbonate rocks have high strontium concentrations and low ^87^Sr/^86^Sr ratios (0.707~0.709), whereas the lower reaches draining a region of clastic sedimentary, magmatic rocks, and metamorphic rocks have lower strontium concentrations and higher ^87^Sr/^86^Sr ratios (0.708~0.910) (Table 2). Figure 4 shows a positive relation with ^87^Sr/^86^Sr and 1/Sr in the dissolved solutes, indicating that the mixture of strontium originating from carbonate and silicate rocks leads to the observed variation in ^87^Sr/^86^Sr ratios for soluble strontium.

## 5. Discussion

### 5.1. Sources of Dissolved Loads

The solutes are derived mainly from atmospheric deposition, anthropogenic contamination, and chemical weathering.

#### 5.1.1. Atmospheric Input

Chloride is the most frequently used proxy to assess the atmospheric input to the chemical composition of dissolved matter in river water [2,27,36,37]. Chloride does not participate in biogeochemical cycling and is comparatively conservative [38]. Chloride concentrations are too low to be detected in rocks apart from evaporates. The annual chloride concentrations of precipitation in Guiyang and Lei Gong Shan were 10.1 μmol/L and 7 μmol/L, respectively [16,39]. The median chloride concentration in rainwater was 8 μmol/L in Guiyang [40]. Xu and Liu (2010) and Jiang et al. (2018) showed that Cl^−^ concentrations from the precipitation for the Xi River were 17.2 and 10.4 μmol/L, respectively [19,26]. Han et al. (2010b) reported an average chloride concentration in the atmosphere of 5.2 μmol/L for Maolan [41]. The atmospheric input of chloride into the river can be calculated by multiplying the evapotranspiration factor by the annual average chloride concentration of precipitation in the basin. The molar ratios of Na^+^/Cl^−^, K^+^/Cl^−^, Ca^2+^/Cl^−^, and Mg^2+^/Cl^−^ in rainwaters were 0.46 ± 0.24, 0.69 ± 0.35, 2.03 ± 1.14, and 0.30 ± 0.16, respectively.

Na-normalized molar ratios of the ions in the precipitation are shown in Table 2. Previous studies reported a minimal contribution of sea salt to water chemistry [17,21]. The average pH of the rainwaters in the Xi River Basin was lower than 4.5, indicating a serious acid rain deposition problem [42]. Most acid rainwaters were SO_4_^2−^-type because of sulfur-rich coal combustion [39]. The SO_4_^2−^/Na^+^ molar ratios in rainwater varied from 1.2 to 24 in the Xi River drainage, implying S enrichment of precipitation relative to sea salt (SO_4_^2−^/Na^+^ = 0.06) [22]. Han et al. (2010b) reported that 40.6 ± 20.7 μmol/L of SO_4_^2−^ in the water body originated from rainwater [42]. The NO_3_^−^/Na^+^ ratios in precipitation ranged from 0.52 to 12.2 in the drainage, pointing to the anthropogenic contribution to the atmosphere (NO_3_^−^/Na^+^ = 0 in sea salt) [22]. The ion concentrations and ratios in rainwater in the city were much higher than those in the countryside, indicating a greater anthropogenic contribution of NOx to the atmosphere near cites.

#### 5.1.2. Anthropogenic Inputs

River water pollution as a result of human activities enters the river water through atmospheric contributions and human emissions, including industrial sewage, fertilizer, and pesticide residues. The characteristics of the Xi River Basin reflect variable natural and anthropogenic processes over a large region and a wide east–west span. There are rich agricultural and mineral resources in the upper reaches, whereas there are high population densities with high urbanization in the lower reaches [22,43]. PO_4_^3−^, NO_3_^−^, K^+^, and Cl^−^ mainly originate from agricultural fertilizers and industrial sewage.

Figure 2 shows the spatial variations in Cl^−^, NO_3_^−^, and SO_4_^2−^ concentrations in the Xi River Basin. Chloride concentrations do not gradually increase with decreasing distances from the sea, suggesting dominant sources other than the influx of marine aerosols, such as halite from evaporite sources and anthropogenic inputs. Halite has not been recorded in the basin, which suggests that recorded Cl^−^ and SO_4_^2−^ originate from other sources. Spatial variations in Cl^−^ indicate that the excess chlorine over atmospheric contributions originate from human activity and are balanced by Na^+^.

**Figure 5:**
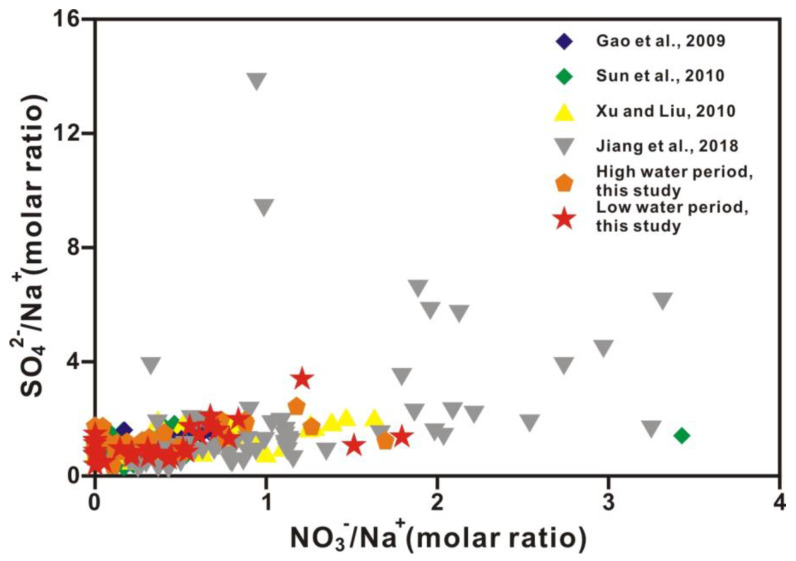
Plots of the molar ratio of SO_4_^2−^/Na^+^ versus the molar ratio of NO_3_^−^/Na^+^ in Xi River drainage. Refs. (Gao et al., 2009; Sun et al., 2010; Xu and Liu, 2010; Jiang et al., 2018) are cited in figure [2,20,22,28].

Figure 5 shows a positive correlation between the SO_4_^2−^/Na^+^ and NO_3_^−^/Na^+^ molar ratios in the Xi River drainage. This relationship indicates that sulfate and nitrate share a common source, possibly anthropogenic. SO_4_^2−^ is derived from gypsum dissolution, sulfide oxidation, and acid deposition. The concentration of NO_3_^−^ is characterized by a large variation range. NO_3_^−^ in river water mainly originates from nitrogen fertilizers used for agriculture. The minor source of nitrate is precipitation. Previous studies reported that gypsum-bearing evaporates were distributed in the Nanpan and Beipan Rivers located in the upper courses [22]. The other source of SO_4_^2−^ in the water body is sulfide oxidation, since coal-bearing sedimentary rocks containing pyrite FeS_2_ are widespread in the Xi River Basin. The positive SO_4_^2−^/Na^+^ and NO^3−^/Na^+^ molar ratios would point to the combustion of S-bearing coal as a significant source of both atmospheric SO_2_ and NOx.

#### 5.1.3. Chemical Weathering Inputs

**Figure 6:**
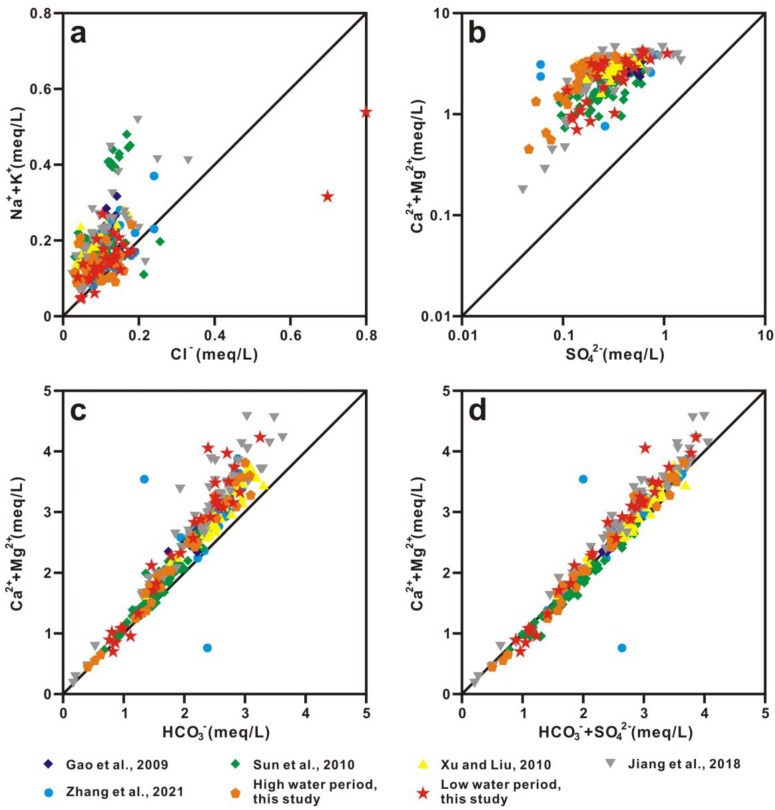
Plots of equivalent ratios of (**a**) (Na^+^ + K^+^) versus Cl^−^, (**b**) (Ca^2+^ + Mg^2+^) versus SO_4_^2−^, (**c**) (Ca^2+^ + Mg^2+^) versus HCO_3_^−^, and (**d**) (Ca^2+^ + Mg^2+^) versus HCO_3_^−^ + SO_4_^2−^ in Xi River drainage waters. Refs. (Gao et al., 2009; Sun et al., 2010; Xu and Liu, 2010; Jiang et al., 2018; Zhang et al.; 2021) are cited in figure [2,10,20,22,28].

Stoichiometric analyses are often used to trace sources of ions dissolved in water bodies. The (Na^+^ + K^+^)/Cl^−^ equivalent ratios in most samples are larger than one, which indicates that Na^+^ and K^+^ mainly originate from sodium and potassium aluminosilicate weathering rather than evaporite weathering (Figure 6a). The excesses Cl^−^ to K^+^ + Na^+^ of Samples X8 (He River) and X15 (Liu River) during the low-water period are derived from residential and industrial wastes (Figure 6a). The (Ca^2^ + Mg^2+^)/(HCO_3_^−^ + SO_4_^2−^) equivalent ratios are close to 1 with high (HCO_3_^−^ + SO_4_^2−^) concentrations, while the (Ca^2^ + Mg^2+^)/HCO_3_^−^ and (Ca^2^ + Mg^2+^)/SO_4_^2−^ equivalent ratios are far from the 1:1 line (Figure 6b–d). This fact suggests that water chemistry is mainly derived from carbonate weathering by carbonic and sulfuric acid (Figure 6b–d). The dissolution of sulfate evaporites (such as gypsum) may be another source. It was estimated that approximately 58.8 μmol/L of riverine SO_4_^2−^ in the upper and middle Xi River was derived from the dissolution of sulfate evaporites [28]. (Ca^2+^ + Mg^2+^)/(HCO_3_^−^ + SO_4_^2−^) equivalent ratios in some samples are lower than one, indicating that extra (HCO_3_^−^ + SO_4_^2−^) is derived from silicate weathering (Figure 6d). The features of the silicate end-member are low Ca^2+^/Na^+^ ratios of 0.01–0.56, Mg^2+^/Na^+^ ratios of 0–0.68, HCO_3_^−^/Na^+^ ratios of 1–3, and high ^87^Sr/^86^Sr ratios of 0.708–0.910 (Table 2) [26]. The features of the carbonate end-member are high Ca^2+^/Na^+^, Mg^2+^/Na^+^, and HCO_3_^−^/Na^+^ ratios of 30–70, 12–28, and 60–140, respectively, and low ^87^Sr/^86^Sr ratios of 0.707–0.709 (Table 2) [26].

**Figure 7:**
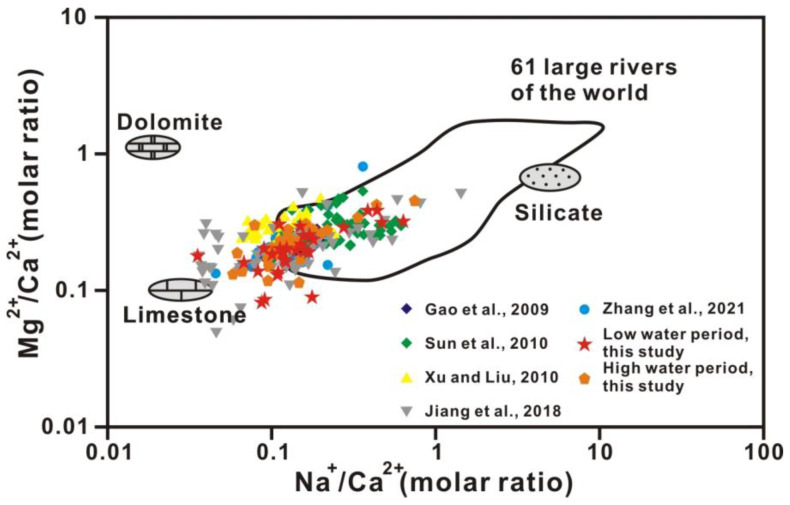
Plots of the molar ratio of Mg^2+^/Ca^2+^ versus the molar ratio of Na^+^/Ca^2+^ (after correction for atmospheric input based on Han et al., 2010b) in Xi River drainage waters [41]. Rock end-members are from Han et al., 2004 [44]. The data distribution area of the 61 large rivers of the world is based on the data compiled by Gaillardet et al., 1999 [11]. Refs. (Gao et al., 2009; Sun et al., 2010; Xu and Liu, 2010; Jiang et al., 2018; Zhang et al.; 2021) are cited in figure [2,10,20,22,28].

**Figure 8:**
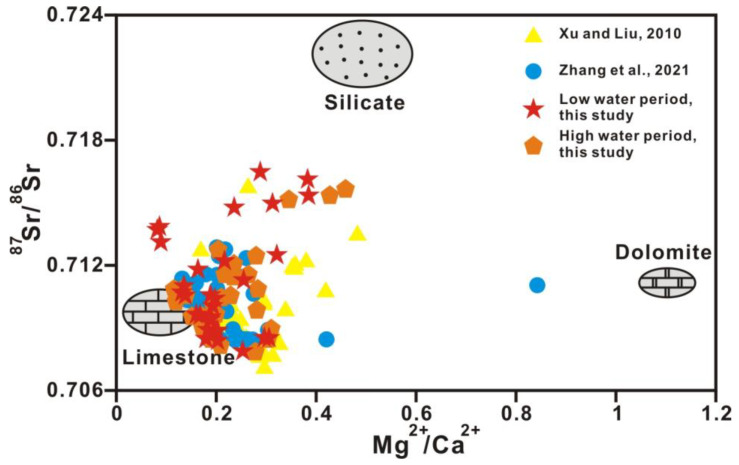
The relationship between the ^87^Sr/^86^Sr and Mg^2+^/Ca^2+^ ratios (after correction for atmospheric input based on Han et al., 2010b) in the river water of the main stream and tributaries [44]. Rock end-members are from Zhang et al., 2021 [10]. Ref. (Xu and Liu, 2010) is cited in figure [22].

The content of strontium, whose chemical properties are stable, is remarkably different in diverse sources. As the strontium isotope ratio is unaffected by material fractionation, dilution, and evaporation effects, the riverine strontium isotopic composition (^87^Sr/^86^Sr) directly reflects the weathering of various source rocks. As carbonates tend to be low in ^87^Rb and high in total strontium, ^87^Sr/^86^Sr ratios of carbonates are commonly interpreted as reflecting the compositions of marine strontium at the time of deposition of carbonate. In contrast, crystalline rocks are often higher in ^87^Rb and lower in total strontium, and here, the ^87^Sr/^86^Sr ratios are a function of Rb content and age. The relationships between Mg^2+^/Ca^2+^ and Na^+^/Ca^2+^ molar ratios and between ^87^Sr/^86^Sr ratios and Mg^2+^/Ca^2+^ molar ratios (after correction for atmospheric input based on Han et al., 2010b) in Xi River water indicate different mixing trends of three end-members, including silicate, limestone, and dolomite (Figure 7 and Figure 8) [41]. In almost all large rivers in the world, chemical weathering contains limestone, dolomite, and silicate weathering. Table 3 shows the ion ratios and strontium isotopic data of silicate, limestone, and dolomite end-members [44]. The features of dolomite end-members are high Mg^2+^/Ca^2+^, Mg^2+^/Sr, Ca^2+^/Sr, and ^87^Sr/^86^Sr ratios of approximately 1.1, 2000, 2000, and 0.711, respectively, and a low Na^+^/Ca^2+^ ratio of approximately 0.02, whereas limestone is characterized by low Mg^2+^/Ca^2+^, Na^+^/Ca^2+^, Mg^2+^/Sr, Ca^2+^/Sr, and ^87^Sr/^86^Sr ratios of approximately 0.1, 0.02, 40–50, 350, and 0.7075, respectively (Figure 7 and Figure 8 and Table 3) [2,44].

**Figure 9:**
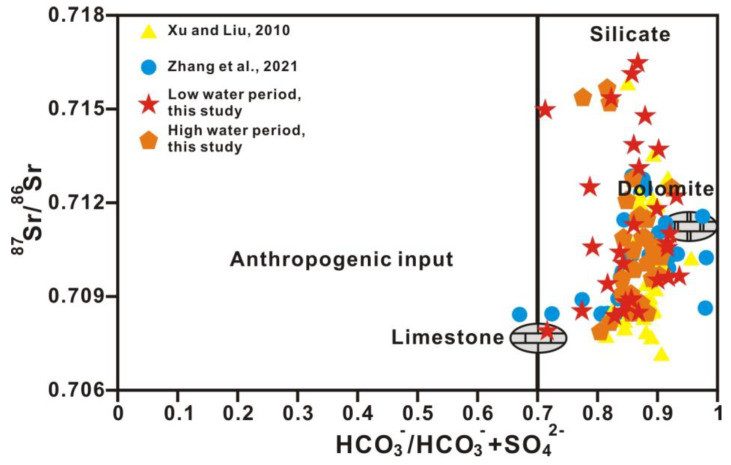
The relationship between ^87^Sr/^86^Sr and HCO_3_^−^/(HCO_3_^−^ + SO_4_^2−^) in river water. Rock end-members are from Zhang et al., 2021 [10]. Ref. (Xu and Liu, 2010) is cited in figure [22].

**Figure 10:**
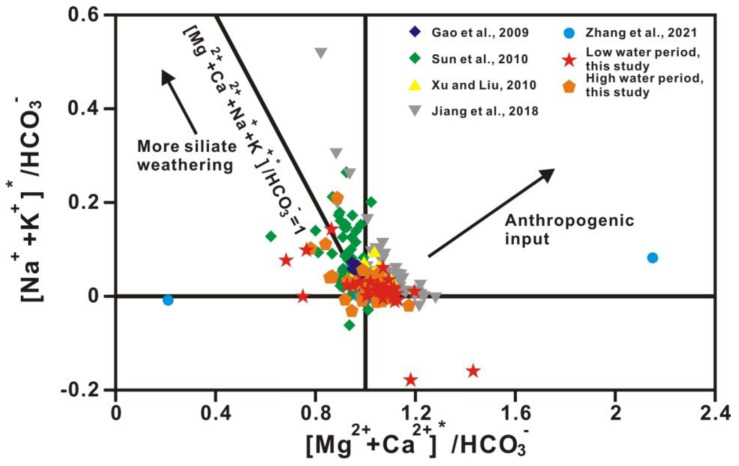
The ionic ratios of silicate and carbonate weathering in Xi River water. [Ca^2+^ + Mg^2+^]* was calculated by subtracting the SO_4_^2−^ equivalence from the total [Ca^2+^ + Mg^2+^] equivalence. [Na^+^ + K^+^]* was calculated by subtracting the Cl^−^ equivalence from the total [Na^+^ + K^+^] equivalence. Refs. (Gao et al., 2009; Sun et al., 2010; Xu and Liu, 2010; Jiang et al., 2018; Zhang et al.; 2021) are cited in figure [2,10,20,22,28].

Figure 7 and Figure 8 show that water chemistry is mainly controlled by limestone weathering, whereas these results cannot identify any anthropogenic activities. Figure 9 shows the relationship between ^87^Sr/^86^Sr ratios and HCO_3_^−^/(HCO_3_^−^ + SO_4_^2−^) equivalent ratios in Xi River water, indicating information on silicate, limestone, and dolomite weathering. The equivalent ratio of HCO_3_^−^/(HCO_3_^−^ + SO_4_^2−^) is greater than 0.7 for all samples in this study, whereas the ratio lower than 0.7 in some samples indicates the influence of anthropogenic activity (Figure 10) [10].

### 5.2. Contributions of the Sources

#### 5.2.1. Calculation Methodology

The inversion model, originally developed by Allègre and Lewin (1989), is commonly used to calculate the chemical weathering contribution [45]. For the estimation, we assumed the following:

– All potassium was derived from silicate weathering;
– Anthropogenic inputs were ignored or classified as atmospheric inputs;
– Evaporite (including halite and gypsum) inputs were ignored.

The model was established on the assumption that dissolved loads originated from three end-members, including atmosphere, carbonate, and silicate. According to the contributions of various end-members, the inversion model was based on a series of mass budget equations of Na-normalized ionic molar ratios (X/Na = Ca/Na, Mg/Na, HCO_3_^−^/Na, Cl/Na and Sr/Na) [Equatioin (1)] and strontium isotopic compositions [Equatioin (2)] of the three end-members (atmosphere, carbonates, and silicates) (Table 2).

(1) $${\left(\frac{X}{\mathrm{Na}}\right)}_{river}=\sum_{i}{\left(\frac{X}{\mathrm{Na}}\right)}_{i}\alpha_{i}\left(\mathrm{Na}\right)\;$$

(2) (S87rS86r)river(SrNa)river=∑i(S87rS86r)i(SrNa)iαi(Na)

where *i* represents the end-member (atmosphere, carbonates, and silicates). The $\alpha_{i}\left(\mathrm{Na}\right)$ represents the mixing proportion of Na in each end-member, and $\underset{i}{\sum }\alpha_{i}\left(\mathrm{Na}\right)\;$= 1. The reason for normalization to Na is that Na^+^ is unaffected by nutrient cycling. K^+^ and SO_4_^2−^ were ignored because they are easily affected by biological activities. The Na-normalized ionic molar ratios and Sr isotopic compositions were used to eliminate the effects of evaporation and discharge [34]. Table 2 shows the chemical compositions of each end-member [10,26,46].

Equations (1) and (2) were weighted by analytical errors of ionic molar ratios and strontium isotopic compositions (10% for elemental concentrations and 0.00002 for ^87^Sr/^86^Sr) to further reduce the error propagation through MATLAB 2022 software. A priori parameters of a series of end-member reservoirs were chosen first. Then, the posterior values that best matched the entire series of model equations were iteratively calculated by the inversion calculation algorithm. A total of 60 samples (30 in the high-water period and 30 in the low-water period) were used in this study. Two hundred model parameters (3*i* × 60$\alpha_{i}\left(\mathrm{Na}\right)$ + 20(X/Na)*_i_*) were solved by successive iterations with equations (6 types × 60 samples = 360 mass balance equations and 60 constraint equations). The mixing proportions of other ions in each reservoir (e.g., $\alpha_{i}\left(\mathrm{Ca}\right)$ and $\alpha_{i}\left(\mathrm{Mg}\right)$) were calculated with the posteriori values of $\alpha_{i}\left(\mathrm{Na}\right)$ for each sample and Na-normalized ionic molar ratios (X/Na)*_i_* of the reservoir (Table 4). Table 4 shows that the features of the end-members in the high- and low-water periods were different. 

**Figure 11:**
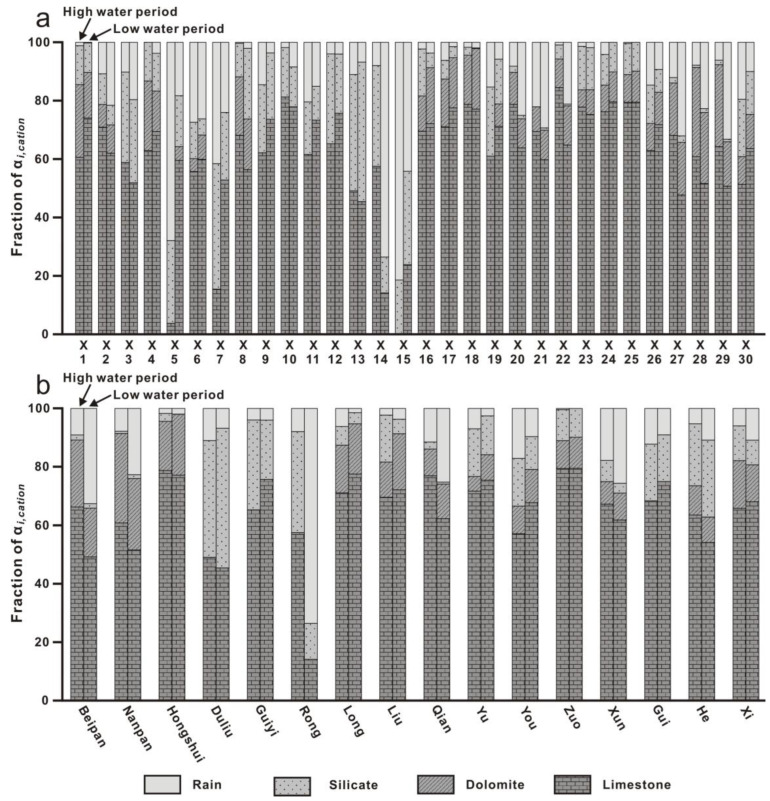
The fraction of total dissolved cations (α*_i,Catin_* = α*_i,Ca_* + α*_i,Na_* + α*_i,Mg_* + α*_i,K_*) from rain, dolomite, limestone, and silicate. (**a**). The fraction of total dissolved cations in all samples. (**b**). The fraction of total dissolved cations in each subbasin.

In the Xi River Basin during the high-water period, the percentage contents of the total of cations from the atmosphere, limestone, dolomite, and silicate were 14.10% (0~81.43%), 60.92% (0~84.48%), 9.17% (0~30.56%), and 15.80% (0~43.00%), respectively (Figure 11a). In the low-water period, these values were 15.62% (0~73.53%), 62.56% (14.16~79.63%), 9.25% (0~24.33%), and 12.57% (0.20~47.79%). Most samples had the largest proportion of cations from limestone, indicating that limestone was the major source of cations owing to the distribution of different types of exposed basement rocks. The contributions of silicate weathering in the high-water period were higher than those in the low-water period. The contributions of dolomite and the atmosphere revealed remarkable variations between high- and low-water periods. Samples X5, X7, X14, and X15 were characterized by abnormally high contributions of precipitation, indicating the influence of anthropogenic activities.

The contributions of carbonate weathering were relatively high in the upper reaches and decreased downstream along the main stream (Figure 11b). Concomitantly, the contributions of silicate weathering increased downstream along the main stream (Figure 11b). These trends are broadly consistent with the predominant geological features in the basin. In the upper reaches, Permian to Triassic carbonate rocks are widely distributed and contribute to carbonate weathering. Precambrian metamorphic rocks and magmatic rocks in the lower reaches enhance the products of silicate weathering. For first-level tributaries, the ratios of the contribution of carbonate/silicate weathering showed an order of Beipan River > Liu River > Yu River > Gui River > He River (Figure 11b). For second-level tributaries, the You River subbasin had a lower contribution of carbonate weathering and a higher contribution of silicate weathering compared to the Zuo River subbasin (Figure 11b). Compared to the Long River subbasin, the Rong River subbasin had a lower contribution of carbonate weathering and a higher contribution of silicate weathering (Figure 11b). These phenomena were consistent with the order of carbonate/silicate area ratios in these subbasins. The spatial variation in the contribution of limestone weathering was different from that in the contribution of dolomite weathering, indicating the inhomogeneous distribution of limestone and dolomite in carbonate rock.

The contributions of the atmosphere were small in most sample settings, except for Samples X5, X7, X14, and X15. Relatively high contributions were recorded in the middle reaches (Qian and Xun Rivers) (Figure 11b), which represent high precipitation in the middle reaches. During the low-water period, relatively high contributions were also recorded in the upper reaches (Nanpan and Beipan Rivers) (Figure 11b), indicating a shorter reaction time with rocks.

#### 5.2.2. Chemical Weathering Rates

The chemical weathering rates of silicates and carbonates were considered a dynamic process. Table 1 shows that the concentrations of NO_3_^−^ were low or even zero in the Xi River Basin. Hence, we only considered that carbonic and sulfuric acid participated in chemical weathering. The silicate weathering rate (SWR) was defined as the sum of cations from silicate weathering:

(3) $$\begin{array}{l} \mathrm{SWR}=\alpha_{sil}\left(\mathrm{Na}\right)\Phi {\mathrm{Na}}_{river}M\left(\mathrm{Na}\right)+\alpha_{sil}\left(K\right)\Phi K_{river}M\left(K\right)+\alpha_{sil}\left(\mathrm{Mg}\right)\Phi {\mathrm{Mg}}_{river}M\left(\mathrm{Mg}\right)+\\ \alpha_{sil}\left(\mathrm{Ca}\right)\Phi {\mathrm{Ca}}_{river}M\left(\mathrm{Ca}\right)+\Phi {\mathrm{SiO}}_{2river}M\left({\mathrm{SiO}}_{2}\right) \end{array}$$

where *Φ* represents the flux of different cations (mol/km^2^/year). $\alpha_{sil}\left(\mathrm{Na}\right)$, $\alpha_{sil}\left(K\right)$, $\alpha_{sil}\left(\mathrm{Mg}\right)$, $\alpha_{sil}\left(\mathrm{Ca}\right)$, and $\Phi {\mathrm{SiO}}_{2river}$ mean the proportions of each cation and SiO_2_ involved in the carbonic and sulfuric acid weathering of silicates. *M*(Na), *M*(K), *M*(Mg), *M*(Ca), and $M\left({\mathrm{SiO}}_{2}\right)$ mean the molar masses of Na, K, Mg, Ca, and SiO_2_.

For the chemical weathering of carbonate, the carbonate weathering rate (CWR) was defined as the sum of cations from carbonate weathering:

(4) $$\mathrm{CWR}=\alpha_{car}\left(\mathrm{Na}\right)\Phi {\mathrm{Na}}_{river}M\left(\mathrm{Na}\right)+\alpha_{car}\left(\mathrm{Mg}\right)\Phi {\mathrm{Mg}}_{river}M\left(\mathrm{Mg}\right)+\alpha_{car}\left(\mathrm{Ca}\right)\Phi {\mathrm{Ca}}_{river}M\left(Ca\right)$$

where $\alpha_{car}\left(\mathrm{Na}\right)$, $\alpha_{car}\left(\mathrm{Mg}\right)$, and $\alpha_{car}\left(\mathrm{Ca}\right)$ mean the proportions of each cation involved in carbonate weathering. 

The limestone weathering rate (LWR) was defined as the total amount of cations from limestone weathering:

(5) $$\mathrm{LWR}=\alpha_{lim}\left(\mathrm{Na}\right)\Phi {\mathrm{Na}}_{river}M\left(\mathrm{Na}\right)+\alpha_{lim}\left(\mathrm{Mg}\right)\Phi {\mathrm{Mg}}_{river}M\left(\mathrm{Mg}\right)+\alpha_{lim}\left(\mathrm{Ca}\right)\Phi {\mathrm{Ca}}_{river}M\left(Ca\right)$$

The dolomite weathering rate (DWR) could be calculated as follows:

(6) DWR=CWR−LWR

We estimated chemical weathering rates through the surface area, runoff, and discharge of the main stream and tributaries, expressed in t/km^2^/year or kg/km^2^/month. Representative samples from the trunk stream and tributaries were used to calculate the chemical weathering contributions in the Xi River Basin. The results are listed in Table 5.

Based on Samples X1 and X2 in the main stream, the SWR values in the Xi River Basin were estimated at 281.38 kg/km^2^/month and 113.65 kg/km^2^/month in the high- and low-water periods, respectively (Appendix A). The SWR varied from one subbasin to another. For tributaries, the SWR values ranged from 1.72 kg/km^2^/month to 1002.18 kg/km^2^/month and from 2.91 kg/km^2^/month to 492.02 kg/km^2^/month, respectively. The SWR values in the high-water period were 0.59- to 16.32-fold of the values in the low-water period. The CWR values in the basin were estimated at 2456.72 kg/km^2^/month and 1409.32 kg/km^2^/month in the high- and low-water periods, respectively. For tributaries, the CWR varied from 186.66 to 6252.48 kg/km^2^/month and from 170.75 to 2084.92 kg/km^2^/month, respectively. The CWR values in the high-water period were 1.09- to 9.00-fold of the values in the low-water period. The LMR was the major component of the CWR. The LWRs in the basin were estimated at 2042.74 kg/km^2^/month and 1222.38 kg/km^2^/month, respectively. For tributaries, the LWR varied from 131.65 to 5248.98 kg/km^2^/month and from 97.42 to 1699.29 kg/km^2^/month, respectively. The LMR values in the high-water period were 1.07- to 9.00-fold those in the low-water period. The DWR in the basin was estimated at 413.98 kg/km^2^/month and 186.94 kg/km^2^/month, respectively. For tributaries, the DWR varied from 0 to 1003.50 kg/km^2^/month and from 0 to 385.63 kg/km^2^/month, respectively. The DWR values in the high-water period were 1.10- to 4.07-fold greater than the values in the low-water period. Seasonal variations in chemical weathering rates were controlled by multiple parameters, including climate (temperature, water discharge, and precipitation) and so on [8,10,20,31,47]. During the high-water period, the warm and humid climate conditions associated with the Asian monsoon enhanced chemical weathering. Higher temperatures can promote the rapid dissolution of minerals [20]. Furthermore, a warm and humid climate can speed up plant degradation, thereby increasing the intensity of chemical weathering by the release of organic acids [10]. The main element dynamics are dominated by water discharge. Hydrological flushing increases the surface area for water-rock interaction and hence accelerates chemical weathering [31,48].

The SWR and CWR values in the upper reaches were lowest in the Xi River Basin. The upper reaches are characterized by relatively low water discharge, temperature, and precipitation. The SWR and CWR values in the Nanpan River were lower than the values in the Beipan River. This phenomenon can be explained by the higher vegetation cover in the Beipan River basin [35,49]. The SWR value increased from 0.03 t/km^2^/year in the upper reaches to 0.59 t/km^2^/year in the middle reaches. The CWR values increased from 2.14 t/km^2^/year in the upper reaches to 32.65 t/km^2^/year in the middle reaches. These phenomena can be explained by the fact that discharge, temperature, and precipitation increased from the upper to the middle reaches. The SWR increased from 0.59 t/km^2^/year in the middle reaches to 2.37 t/km^2^/year in the lower reaches, whereas the CWR decreased downstream along the main stream with values from 32.65 t/km^2^/year to 23.20 t/km^2^/year. Carbonate/silicate area ratios decreased from the middle to lower reaches. For the first-level tributaries, the SWR ranged from 0.12 t/km^2^/year to 8.97 t/km^2^/year, with an order of He River > Gui River > Yu River > Liu River > Beipan River (Table 5). The CWR ranged from 6.09 t/km^2^/year to 34.12 t/km^2^/year, with an order of He River > Gui River > Liu River > Yu River > Beipan River (Table 5). The spatial variation in the LWR was consistent with that in the CWR, whereas the spatial variation in the DWR was inconsistent. This phenomenon can be explained by the inhomogeneous distribution of limestone and dolomite in carbonate rock.

#### 5.2.3. CO_2_ Consumption Rate

The proportion of sulfuric acid in different rock weathering was an important factor in calculating the CO_2_ flux absorbed by the chemical weathering of silicates. When we assumed that all SO_4_^2−^ derived from gypsum coexisting with carbonates, the carbonic acid weathering of silicates (CSW) was equivalent to the CO_2_ flux consumed by silicate weathering. Sulfuric acid played a significant role in chemical weathering processes due to anthropogenic activities. There was no CO_2_ consumption during the sulfuric acid weathering of silicate. The CSW value was defined as the sum total of cations from the carbonic acid weathering of silicate:

(7) $$\mathrm{CSW}=\alpha_{sil}\left(\mathrm{Na}\right)\Phi {\mathrm{Na}}_{river}+\alpha_{sil}\left(K\right)\Phi K_{river}+2\alpha_{sil}\left(\mathrm{Mg}\right)\Phi {\mathrm{Mg}}_{river}+\;2\alpha_{sil}\left(\mathrm{Ca}\right)\Phi {\mathrm{Ca}}_{river}-\delta *2\Phi {\mathrm{SO}}_{4river}$$

where *δ* represents the adjustment coefficient of sulfuric acid with a value from 0 to 1. The proportion of the sulfuric acid weathering of carbonate and silicate was equivalent to the contribution rate of carbonate and silicate to the total dissolved cations. 

CO_2_ consumption involved in carbonate weathering by carbonic acid (CCW) could be expressed as:

(8) $$\mathrm{CCW}={\mathrm{CO}}_{2car}=0.5\times ({\left[{\mathrm{HCO}}_{3}\right]}_{river}-\;\mathrm{CSW})\;$$

Furthermore, CO_2_ generated by the sulfuric acid weathering of carbonate (SCW) could be expressed as:

(9) $$\mathrm{SCW}=\left(1-\delta \right)\Phi {\mathrm{SO}}_{4river}$$

The corresponding CO_2_ production due to the sulfuric acid weathering of limestone (SLW) was expressed as follows:

(10) SLW=β∗SCW

The corresponding CO_2_ production due to the sulfuric acid weathering of dolomite (SDW) was expressed as follows:

(11) SDW=(1−β)∗SCW

where *β* represents the adjustment coefficient of limestone in carbonate with values from 0 to 1. *β* was calculated by the dissolved cation contribution ratio of limestone and dolomite, which could be further applied to calculate the related CO_2_ flux of limestone and dolomite.

The corresponding CO_2_ consumed by the carbonic acid weathering of limestone (CLW) could be calculated as follows:

(12) CLW=β∗CCW

The corresponding CO_2_ consumed by the carbonic acid weathering of dolomite (CDW) could be calculated as follows:

(13) CDW=CCW−CLW

In this study, the CO_2_ flux consumed by chemical weathering in the basin was estimated at 189.79 × 10^9^ mol/year based on Samples X1 and X2. The CO_2_ fluxes during the high- and low-water periods were 124.03 × 10^9^ and 65.76 × 10^9^ mol/year, respectively, accounting for 65.35% and 34.65% of the total flux. The water discharge in the basin during the high-water period was 2.63-fold that during the low-water period. The carbon sink was primarily controlled by the water cycle. The contributions of each end-member were slightly different in strength during different periods. The CO_2_ fluxes consumed by silicate weathering during the high- and low-water periods were 24.04 × 10^9^ mol/year and 9.38 × 10^9^ mol/year, respectively, accounting for 12.67% and 4.94% of the total flux. The CO_2_ fluxes consumed by carbonate weathering were 99.99 × 10^9^ mol/year and 56.38 × 10^9^ mol/year, respectively, accounting for 52.68% and 29.71% of the total flux. The CO_2_ fluxes consumed by limestone weathering were 79.81 × 10^9^ mol/year and 47.67 × 10^9^ mol/year, respectively, accounting for 42.05% and 25.11% of the total flux. The CO_2_ fluxes consumed by dolomite weathering were 20.18 × 10^9^ mol/year and 8.71 × 10^9^ mol/year, respectively, accounting for 10.63% and 4.59% of the total flux.

The total CO_2_ fluxes consumed by silicate and carbonate weathering in the basin were estimated at 33.42 × 10^9^ mol/year and 156.37 × 10^9^ mol/year, respectively, accounting for 0.38% and 1.27% of the global CO_2_ consumption fluxes (8.7 × 10^12^ and 12.3 × 10^12^ mol/year [13]) (Table 5). For first-level tributaries, CO_2_ fluxes consumed by chemical weathering ranged from 51.32 × 10^9^ mol/year in the Yu River to 2.80 × 10^9^ mol/year in the Beipan River. The CO_2_ fluxes consumed by chemical weathering in the Beipan, Liu, Yu, Gui, and He River subbasins accounted for 1.47%, 17.03%, 27.04%, 6.96%, and 5.96% of the total CO_2_ consumption flux in the Xi River Basin, respectively. The CO_2_ fluxes consumed by silicate weathering ranged from 13.34 × 10^9^ mol/year in the Yu River to 0.08 × 10^9^ mol/year in the Beipan River. The CO_2_ fluxes consumed by silicate weathering in the Beipan, Liu, Yu, Gui, and He River subbasins accounted for 0.24%, 22.63%, 39.90%, 11.15%, and 12.76% of the total CO_2_ consumption flux, respectively. The CO_2_ fluxes consumed by carbonate weathering ranged from 37.99 × 10^9^ mol/year in the Yu River to 2.72 × 10^9^ mol/year in the Beipan River. The CO_2_ fluxes consumed by carbonate weathering in the Beipan, Liu, Yu, Gui, and He River subbasins accounted for 1.74%, 15.84%, 24.29%, 6.07%, and 4.51% of the total CO_2_ consumption fluxes, respectively. The CO_2_ fluxes consumed by limestone weathering ranged from 35.09 × 10^9^ mol/year in the Yu River to 2.02 × 10^9^ mol/year in the Beipan River. The CO_2_ fluxes consumed by limestone weathering in the Beipan, Liu, Yu, Gui, and He River subbasins accounted for 1.59%, 16.23%, 27.53%, 7.44%, and 4.74% of the total CO_2_ consumption fluxes, respectively. The CO_2_ fluxes consumed by dolomite weathering ranged from 4.07 × 10^9^ mol/year in the Liu River to 0 mol/year in the Gui River. The CO_2_ fluxes consumed by dolomite weathering in the Beipan, Liu, Yu, Gui, and He River subbasins accounted for 2.40%, 14.10%, 10.02%, 0%, and 1.01% of the total CO_2_ consumption fluxes, respectively.

#### 5.2.4. Sulfuric Acid as Weathering Agent

**Figure 12:**
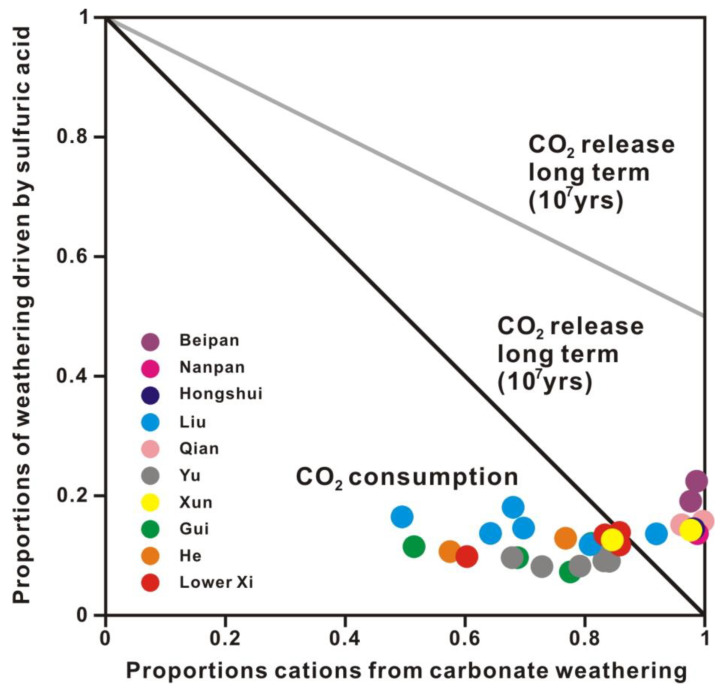
Diagram showing the effect of chemical weathering on atmospheric CO_2_. The long-term and short-term consequences are considered. Proportions of weathering driven by sulfuric acid are calculated by SO_4_^2−^/Σ^+^_weathering_, and proportions of cations from carbonate weathering are calculated by Σ^+^_carbonate_/Σ^+^_weathering_ (equivalent ratio). The insert is an expanded view of the Xi River.

The carbon budget of chemical weathering consisted of CO_2_ consumption by chemical weathering and CO_2_ emission by sulfuric acid weathering. The proportions of sulfuric acid weathering were plotted against the proportions of cations from carbonate weathering to research the effects of sulfuric acid weathering on atmospheric CO_2_ (Figure 12) [2,6]. The proportions of cations produced by carbonate weathering ranged from 49.45% to 99.67% (Figure 12). The proportions of sulfuric acid weathering ranged from 7.26% to 22.42% (Figure 12). The CO_2_ budget by chemical weathering was dramatically influenced by sulfuric acid. The participation of sulfuric acid in carbonate weathering greatly promotes chemical weathering but reduces the CO_2_ consumption flux.

Samples in the upper reaches and middle trunk stream were characterized by higher proportions of cations from carbonate weathering and higher proportions of sulfuric acid weathering, indicating a high sulfuric acid weathering contribution to atmospheric CO_2_ emissions. The upper and middle reaches are carbon sources on a timescale of 10^7^ years. Based on Samples X6 and X20 in the Xun River, the CO_2_ flux produced by sulfuric acid weathering was estimated at 36.06 × 10^9^ mol/year in the upper and middle reaches. However, the CO_2_ flux consumed by silicate weathering was 25.43 × 10^9^ mol/year. Therefore, the upper and middle reaches with a widespread distribution of carbonate were net carbon sources on a timescale of 10^7^ years with a net released CO_2_ flux of 10.63 × 10^9^ mol/year. For tributaries, the Beipan River was a carbon source with a net released CO_2_ flux of 0.90 × 10^9^ mol/year. However, the Yu and Liu Rivers were carbon sinks with net consumed CO_2_ fluxes of 8.48 × 10^9^ mol/year and 3.08 × 10^9^ mol/year, respectively.

Samples in the lower reaches were characterized by lower proportions of cations from carbonate weathering, which acted as carbon sinks (Figure 12). The Gui and He Rivers were carbon sinks with net consumed CO_2_ fluxes of 2.41 × 10^9^ mol/year and 2.85 × 10^9^ mol/year, respectively. Based on Samples X1 and X2 in the main stream, the CO_2_ flux produced by sulfuric acid weathering was estimated at 30.00 × 10^9^ mol/year in the Xi River Basin, accounting for 13.65% of the total CO_2_ consumption. The CO_2_ flux consumed by silicate weathering was 33.42 × 10^9^ mol/year. Therefore, the Xi River Basin was a carbon sink with a net consumed CO_2_ flux of 3.42 × 10^9^ mol/year.

## 6. Conclusions

We present new major ion and Sr isotope ratio data on the chemical evolution of the Xi river in relation to chemical weathering processes in the drainage basin. An inversion model was used to estimate the chemical weathering rates and CO_2_ consumption fluxes in the Xi River Basin at monthly and annual scales. The primary conclusions were as follows:

1. The water in the Xi River drainage is slightly alkaline with average pH values of 8.00 and 7.87 during the high- and low-water periods, respectively. The water was the HCO_3_—Ca/Mg type. The concentrations of Ca^2+^, Mg^2+^, HCO_3_^−^, and Sr decreased downstream along the main stream of the Xi River, whereas the ^87^Sr/^86^Sr ratios increased downstream. Spatial variations were consistent with the lithologic spatial distribution. Carbonates were most abundant in the upper courses, while more silicates appeared in the lower courses. Most major ion concentrations in the high-water period were in general lower than those in the low-water period. Seasonal variations were dominantly controlled by the water discharge, although a larger area of water-rock interaction could enhance chemical weathering. Variations in chemical weathering rates were controlled by climate (temperature, water discharge, and precipitation), vegetation, and so on. Higher temperatures, increased reactive mineral surface areas, and organic acids can accelerate chemical weathering.
2. In the Xi River Basin, the SWR value was estimated at 2.37 t/km^2^/year, with values of 281.38 kg/km^2^/month and 113.65 kg/km^2^/month during the high- and low-water periods, respectively. The CWR value was estimated at 23.20 t/km^2^/year, with values of 2456.72 kg/km^2^/month and 1409.32 kg/km^2^/month, respectively. The LWR value was estimated at 19.59 t/km^2^/year, with values of 2042.74 kg/km^2^/month and 1222.38 kg/km^2^/month, respectively. The DWR value was estimated at 3.61 t/km^2^/year with values of 413.98 kg/km^2^/month and 186.94 kg/km^2^/month, respectively.
3. The SWR values increased from 0.03 t/km^2^/year in the upper reaches to 2.37 t/km^2^/year in the lower reaches. The CWR values increased from 2.14 t/km^2^/year in the upper reaches to 32.65 t/km^2^/year in the middle reaches and then decreased to 23.20 t/km^2^/year in the lower reaches. The chemical weathering rates varied from one subbasin to another. The spatial variations in chemical weathering rates were controlled by lithology, vegetation, climate, and soil conditions.
4. The CO_2_ flux consumed by chemical weathering was 189.79 × 10^9^ mol/year in Xi River drainage. The CO_2_ fluxes consumed by carbonate and silicate weathering were 156.37 × 10^9^ and 33.42 × 10^9^ mol/year, respectively, accounting for 1.27% and 0.38% of the global CO_2_ consumption fluxes. The CO_2_ consumption fluxes by limestone and dolomite weathering were 127.48 × 10^9^ and 28.89 × 10^9^ mol/year, respectively. Sulfuric acid played a significant role in the CO_2_ budget by chemical weathering. The CO_2_ flux produced by sulfuric acid weathering was estimated at 30.00 × 10^9^ mol/year in the basin. The upper and middle reaches were net carbon sources on a timescale of 10^7^ years with a net released CO_2_ flux of 10.63 × 10^9^ mol/year. However, the Xi River Basin was a CO_2_ sink with a net consumed CO_2_ flux of 3.42 × 10^9^ mol/year.

## Figures and Tables

**Table 1:** Basic parameters and chemical compositions of the main ions at each sampling point of the main stream and tributaries of the Xi River.

River Name	Sample No.	Date	pH	K^+^	Na^+^	Ca^2+^	Mg^2+^	Cl^−^	SO_4_^2−^	HCO_3_^−^	NO_3_^−^	SiO_2_	Sr (μmol/L)	^87^Sr/^86^Sr	TDS (mg/L)	TZ^+^ (μeq/L)	TZ^−^ (μeq/L)	NICB (%)
				μmol/L														
Lower Xi	X1	July 2019	7.88	9	116	674	209	70	135	1611	0	140	1.507	0.7090	157.11	1891	1951	−1.56
Lower Xi	X2		8.03	0	94	745	153	98	117	1429	26	142	0.619	0.7128	147.65	1890	1787	2.80
He	X3		8.18	21	113	821	197	148	137	1785	21	269	1.164	0.7115	185.72	2170	2228	−1.32
Lower Xi	X4		8.05	33	108	796	224	64	139	1724	0	132	1.234	0.7099	169.69	2181	2066	2.71
Lower Xi	X5		7.98	19	98	689	146	97	111	1352	0	108	0.791	0.7105	137.11	1787	1671	3.35
Xun	X6		7.88	21	122	1045	204	85	146	2215	0	122	1.105	0.7101	209.79	2641	2592	0.94
Gui	X7		7.94	29	94	490	139	72	111	1193	12	176	0.468	0.7109	123.52	1381	1499	−4.10
He	X8		8.24	19	119	987	231	134	145	2173	202	106	0.687	0.7115	218.62	2574	2799	−4.19
Gui	X9		8.01	0	96	616	142	104	89	1456	19	132	0.997	0.7106	140.41	1612	1757	−4.30
Gui	X10		7.94	3	116	547	145	62	103	1374	16	154	0.813	0.7116	134.28	1503	1658	−4.90
Gui	X11		7.93	0	121	525	147	160	54	1307	16	155	0.717	0.7125	128.19	1465	1591	−4.12
Guyi	X12		7.51	0	117	157	70	31	46	408	12	134	0.458	0.7157	49.84	571	543	2.51
Duliu	X13		7.45	19	87	245	84	36	68	621	0	146	0.441	0.7152	69.01	764	793	−1.86
Rong	X14		7.48	0	90	198	83	36	76	525	0	157	0.418	0.7154	62.00	652	713	−4.47
Liu	X15		7.92	12	105	787	186	65	179	1557	5	98	0.923	0.7121	159.49	2063	1985	1.93
Liu	X16		7.73	0	98	724	158	49	128	1468	0	125	0.892	0.7116	146.08	1862	1773	2.45
Long	X17		7.89	27	101	1504	287	88	248	2945	119	88	1.659	0.7085	289.66	3710	3648	0.84
Hongshui	X18		7.98	32	138	1326	248	106	192	2628	105	89	1.969	0.7088	257.77	3318	3223	1.45
Yu	X19		7.92	65	178	1165	137	181	154	2150	21	71	0.788	0.7109	214.44	2847	2660	3.40
Xun	X20		7.91	0	104	853	163	122	141	1676	33	123	0.926	0.7091	169.95	2136	2113	0.54
Qian	X21		8.03	0	124	1231	213	78	219	2310	0	91	1.236	0.7091	227.37	3012	2826	3.19
Qian	X22		8.17	29	112	1084	166	99	196	2070	5	108	0.987	0.7096	206.44	2641	2566	1.44
Yu	X23		8.20	32	131	1294	157	151	131	2530	0	89	0.898	0.7103	237.40	3065	2943	2.03
Yu	X24		8.15	5	101	1402	198	131	154	2713	41	134	0.993	0.7106	258.86	3306	3193	1.74
Zuo	X25		8.09	0	92	1441	194	138	171	2495	81	109	0.789	0.7109	249.48	3362	3056	4.77
You	X26		8.21	0	204	1320	228	113	175	2860	103	102	0.841	0.7096	270.74	3300	3426	−1.87
Beipan	X27		8.29	27	166	1580	331	41	327	3005	122	98	2.810	0.7082	305.61	4015	3822	2.46
Nanpan	X28		8.47	15	117	1383	420	58	204	3079	148	78.2	1.749	0.7085	292.01	3738	3693	0.61
Beipan	X29		8.51	31	177	1372	382	46	346	2846	155	103	2.915	0.7079	293.57	3716	3739	−0.31
You	X30		8.07	0	159	1368	275	81	166	3095	78	124	0.892	0.7097	284.86	3445	3586	−2.01
Lower Xi	X1	October 2019	7.66	11	177	1074	209	159	201	2144	0	112	1.107	0.7101	214.89	2754	2704	0.91
Lower Xi	X2		7.69	9	148	1188	225	135	120	2163	54	116	1.347	0.7095	215.29	2985	2593	7.03
He	X3		7.88	8	121	418	120	101	73	953	52	136	0.387	0.7165	102.82	1206	1252	−1.88
Lower Xi	X4		7.81	9	142	950	188	128	174	1786	0	110	0.972	0.7104	182.90	2427	2262	3.52
Lower Xi	X5		7.76	10	113	701	153	97	55	1489	6	89	0.661	0.7122	139.91	1830	1701	3.67
Xun	X6		7.96	10	146	1210	228	133	255	2273	81	133	1.358	0.7094	238.51	3034	2997	0.61
Gui	X7		7.53	11	102	533	127	84	85	1240	22	174	0.436	0.7148	125.76	1433	1517	−2.84
He	X8		7.94	21	294	1703	323	698	315	2392	445	134	0.767	0.7106	319.98	4368	4164	2.39
Gui	X9		7.36	0	94	496	47	72	74	987	21	172	0.365	0.7131	104.65	1180	1229	−2.04
Gui	X10		7.46	5	45	408	38	49	62	764	81	177	0.308	0.7139	88.38	941	1018	−3.92
Gui	X11		7.51	0	46	438	39	47	61	1111	36	175	0.321	0.7137	107.52	1000	1315	−13.62
Guyi	X12		7.63	0	102	254	96	39	69	824	32	128	0.468	0.7161	82.67	801	1032	−12.57
Duliu	X13		7.50	18	185	388	121	89	162	804	0	100	0.456	0.7150	97.15	1222	1217	0.23
Rong	X14		7.45	0	137	306	117	53	93	863	29	129	0.506	0.7154	91.22	983	1131	−7.02
Liu	X15		7.91	29	510	802	257	799	197	1459	0	107	0.779	0.7125	193.82	2656	2653	0.06
Liu	X16		7.88	0	130	724	185	82	125	1538	19	103	0.795	0.7113	152.50	1948	1890	1.52
Long	X17		7.94	0	61	1482	267	83	207	2729	74	85	1.369	0.7085	266.09	3559	3301	3.77
Hongshui	X18		7.83	4	148	1552	316	112	293	2828	124	97	3.218	0.7084	291.35	3887	3651	3.13
Yu	X19		8.03	18	148	1282	175	171	105	2431	66	82	0.666	0.7110	233.07	3080	2879	3.37
Xun	X20		7.95	5	146	1371	252	138	211	2512	89	79	2.031	0.7089	253.08	3397	3161	3.61
Qian	X21		7.90	4	155	1308	263	133	228	2509	0	195	2.271	0.7088	253.63	3299	3097	3.15
Qian	X22		8.28	5	170	1320	268	140	214	2546	0	135	2.237	0.7089	252.22	3351	3113	3.68
Yu	X23		7.89	16	155	1354	179	184	121	2640	0	56	0.698	0.7107	245.16	3237	3065	2.72
Yu	X24		7.99	3	120	1358	189	151	117	2560	37	68	0.749	0.7106	240.86	3217	2982	3.79
Zuo	X25		8.01	7	129	996	164	123	109	1935	69	125	0.662	0.7118	191.61	2456	2344	2.33
You	X26		8.05	0	100	1357	219	68	95	2803	51	167	0.786	0.7096	257.63	3252	3114	2.18
Beipan	X27		8.38	13	207	1341	397	133	367	2511	138	98	3.309	0.7085	275.93	3697	3514	2.53
Nanpan	X28		8.43	19	188	1620	495	147	304	3250	134	128	2.944	0.7085	330.46	4437	4140	3.46
Beipan	X29		8.48	14	255	1583	401	104	536	2704	171	88	4.975	0.7079	315.40	4236	4053	2.22
You	X30		7.96	0	177	1422	240	106	131	2918	0	156	0.856	0.7096	270.37	3501	3285	3.18

**Table 2:** Parameters of each model end-member.

End Member	Ca^2+^/Na^+^	Mg^2+^/Na^+^	HCO_3_^−^/Na^+^	Cl^−^/Na^+^	1000 * Sr/Na^+^	^87^Sr/^86^Sr
Marine aerosol [33]	0.022	0.12	0.004	0.19	1.16	0.709
Rain in high-water period [35]	3.83	1.08	23.14	1.41	16.51	0.709 [36]
Rain in low-water period [35]	1.66	0.30	13.58	0.61	9.69	0.709 [36]
Carbonate [33]	30–70	12–28	60–140	0.001	50–100	0.707–0.709
Silicate [33]	0.01–0.56	0–0.68	1–3	0.001	1–175	0.708–0.910

**Table 3:** Molar ratios of the river end-members [44].

End Member	Mg^2+^/Ca^2+^	Na^+^/Ca^2+^	Mg^2+^/Sr	Ca^2+^/Sr	Na^+^/Sr	^87^Sr/^86^Sr	HCO_3_^−^/(HCO_3_^−^ + SO_4_^2−^)
Limestone	~0.1	~0.02	40–50	~350	>10	~0.7075	~0.7
Dolomite	~1.1	~0.02	~2000	~2000	>100	~0.711	~0.9
Silicate	0.4–0.8	~5	~200	~200	>700	>0.715	0.8–0.9

**Table 4:** End-member parameters of the model.

End Member	Ca^2+^/Na^+^	Mg^2+^/Na^+^	HCO_3_^−^/Na^+^	Cl^−^/Na^+^	1000 * Sr/Na^+^	^87^Sr/^86^Sr
High-water period						
Rain	3.83	1.08	23.14	1.41	16.51	0.7090
Carbonate	43.44	12.00	64.55	0.001	50	0.7083
Silicate	0.56	0.02	3.00	0.001	1.00	0.7108
Low-water period						
Rain	1.66	0.30	13.58	0.61	9.69	0.7090
Carbonate	52.41	12.00	78.21	0.001	50	0.7090
Silicate	0.56	0.06	3.00	0.001	1.00	0.7225

**Table 5:** Chemical weathering rates and CO_2_ consumption for the main stream and main tributaries of the Xi River.

River	Chemical Weathering Rates t/km^2^/Year				CO_2_ Consumption											
					Carbonic Acid 10^9^ mol/Year				Sulfuric Acid−10^9^ mol/Year			Carbonate Weathering 10^9^ mol/Year	Limestone Weathering 10^9^ mol/Year	Dolomite Weathering 10^9^ mol/Year	Silicate Weathering 10^9^ mol/Year	Total 10^9^ mol/Year
	SWR	CWR	LWR	DWR	CCW	CLW	CDW	CSW	SCW	SLW	SDW					
Beipan	0.12	6.09	4.74	1.35	3.70	2.75	0.95	0.08	0.98	0.73	0.25	2.72	2.02	0.69	0.08	2.80
Nanpan	0.03	2.14	1.53	0.62	3.00	2.01	0.99	0.04	0.48	0.32	0.16	2.52	1.69	0.83	0.04	2.56
Hongshui	0.47	29.11	24.27	4.85	82.28	66.40	15.88	1.72	14.11	11.34	2.77	68.17	55.05	13.12	1.72	69.89
Yu	3.62	21.71	20.31	1.40	42.85	39.62	3.23	13.34	4.86	4.52	0.34	37.99	35.09	2.89	13.34	51.32
You	3.88	20.51	18.05	2.46	22.04	18.89	3.15	6.66	2.36	2.02	0.34	19.69	16.87	2.81	6.66	26.35
Zuo	3.43	35.14	31.90	3.24	21.91	19.52	2.39	4.77	2.95	2.63	0.32	18.96	16.89	2.07	4.77	23.73
Liu	3.10	23.77	20.52	3.25	29.24	24.42	4.82	7.56	4.48	3.74	0.74	24.76	20.69	4.07	7.56	32.32
Rong	2.99	5.85	5.85	0.00	4.69	4.69	0.00	2.42	1.21	1.21	0.00	3.48	3.48	0.00	2.42	5.90
Long	2.72	48.58	40.83	7.74	17.56	14.29	3.27	1.67	2.91	2.37	0.54	14.65	11.92	2.72	1.67	16.32
Duliu	5.96	7.63	7.63	0.00	2.16	2.16	0.00	2.47	0.59	0.59	0.00	1.57	1.57	0.00	2.47	4.04
Guyi	2.92	9.91	9.91	0.00	1.18	1.18	0.00	0.56	0.24	0.24	0.00	0.94	0.94	0.00	0.56	1.50
Qian	0.59	32.65	29.28	3.37	153.73	134.70	19.03	3.51	28.43	24.94	3.49	125.29	109.76	15.54	3.51	128.80
Xun	2.05	27.15	24.67	2.49	220.00	196.44	23.56	25.43	36.06	32.07	3.98	183.94	164.37	19.57	25.43	209.37
Gui	4.48	19.93	19.93	0.00	10.81	10.81	0.00	3.73	1.32	1.32	0.00	9.49	9.49	0.00	3.73	13.21
He	8.97	34.12	29.45	4.67	8.47	7.24	1.22	4.26	1.41	1.20	0.21	7.06	6.04	1.01	4.26	11.32
Low Xi	2.37	23.20	19.59	3.61	186.37	151.83	34.54	33.42	30.00	24.34	5.65	156.37	127.48	28.89	33.42	189.79

## Data Availability

The data supporting reported results can be found in the tables in this text and Appendix A.

## Appendix A

**Table:**

River	Period	Area (km^2^)	Runoff (mm/Year)	Discharge (km^3^/Year)	Chemical Weathering Rates kg/km^2^/Month				CO_2_ Consumption											
									Carbonic Acid 10^9^ mol/Month				Sulfuric Acid −10^9^ mol/Month			Carbonate Weathering 10^9^ mol/Month	Limestone Weathering 10^9^ mol/Month	Dolomite Weathering 10^9^ mol/Month	Silicate Weathering 10^9^ mol/Month	Total 10^9^ mol/Month
					SWR	CWR	LWR	DWR	CCW	CLW	CDW	CSW	SCW	SLW	SDW					
Beipan	High-water period	26,557	67.40	1.79	14.49	739.99	574.68	165.31	0.43	0.32	0.11	0.01	0.10	0.07	0.03	0.33	0.25	0.09	0.01	0.34
Nanpan		56,809	17.60	1.00	1.72	186.66	131.65	55.01	0.26	0.17	0.09	0.00	0.03	0.02	0.01	0.22	0.15	0.07	0.00	0.22
Hongshui		137,719	272.37	37.51	74.23	2767.40	2344.94	422.47	8.08	6.64	1.44	0.27	1.18	0.97	0.21	6.90	5.67	1.23	0.27	7.17
Yu		89,677	342.12	30.68	486.48	2710.88	2557.84	153.05	5.41	5.06	0.36	1.77	0.65	0.61	0.04	4.76	4.44	0.32	1.77	6.53
You		38,612	342.64	13.23	537.50	2525.19	2224.64	300.55	2.82	2.42	0.40	0.92	0.32	0.28	0.05	2.50	2.14	0.36	0.92	3.42
Zuo		32,068	492.70	15.80	485.16	4784.88	4351.72	433.17	2.94	2.63	0.31	0.68	0.41	0.37	0.04	2.53	2.26	0.27	0.68	3.22
Liu		57,173	604.31	34.55	479.01	2973.24	2608.95	364.29	3.64	3.10	0.54	1.18	0.54	0.46	0.08	3.09	2.63	0.46	1.18	4.27
Rong		21,585	706.51	15.25	428.37	876.93	876.93	0.00	0.49	0.49	0.00	0.35	0.14	0.14	0.00	0.35	0.35	0.00	0.35	0.70
Long		16,449	599.43	9.86	392.94	6252.48	5248.98	1003.50	2.30	1.87	0.43	0.24	0.39	0.32	0.07	1.91	1.55	0.36	0.24	2.15
Duliu		11,326	702.81	7.96	636.54	889.92	889.92	0.00	0.27	0.27	0.00	0.27	0.06	0.06	0.00	0.21	0.21	0.00	0.27	0.49
Guyi		5098	890.55	4.54	386.76	1140.28	1140.28	0.00	0.12	0.12	0.00	0.07	0.03	0.03	0.00	0.09	0.09	0.00	0.07	0.16
Qian		198,005	490.29	97.08	85.88	3862.78	3515.31	347.47	17.47	15.59	1.88	0.50	3.31	2.96	0.36	14.15	12.63	1.52	0.50	14.65
Xun		308,271	532.29	164.09	284.64	3040.41	2787.84	252.57	24.81	22.42	2.39	3.59	3.70	3.33	0.37	21.11	19.09	2.02	3.59	24.70
Gui		19,288	770.43	14.86	622.68	2624.59	2624.59	0.00	1.45	1.45	0.00	0.52	0.17	0.17	0.00	1.27	1.27	0.00	0.52	1.80
He		11,536	720.35	8.31	1002.18	4121.33	3609.23	512.09	1.13	0.97	0.16	0.48	0.16	0.14	0.02	0.97	0.83	0.14	0.48	1.45
Low Xi		353,100	492.04	173.74	281.38	2456.72	2042.74	413.98	20.00	15.97	4.04	4.01	3.34	2.66	0.68	16.66	13.30	3.36	4.01	20.67
Beipan	Low-water period	26,557	32.38	0.86	5.69	274.24	214.76	59.49	0.18	0.14	0.05	0.00	0.06	0.05	0.02	0.12	0.09	0.03	0.00	0.12
Nanpan		56,809	16.02	0.91	2.91	170.75	122.77	47.98	0.24	0.17	0.08	0.00	0.05	0.03	0.01	0.20	0.13	0.06	0.00	0.20
Hongshui		137,719	173.83	23.94	4.55	2084.92	1699.29	385.63	5.64	4.43	1.21	0.01	1.17	0.92	0.25	4.47	3.51	0.96	0.01	4.48
Yu		89,677	102.70	9.21	117.64	907.60	827.66	79.94	1.73	1.55	0.18	0.45	0.16	0.14	0.02	1.57	1.41	0.16	0.45	2.02
You		38,612	102.82	3.97	109.42	892.65	783.34	109.32	0.85	0.73	0.12	0.19	0.07	0.06	0.01	0.78	0.67	0.11	0.19	0.97
Zuo		32,068	147.81	4.74	86.62	1071.38	964.83	106.55	0.71	0.62	0.08	0.11	0.08	0.07	0.01	0.63	0.55	0.07	0.11	0.74
Liu		57,173	174.21	9.96	37.54	988.47	810.55	177.92	1.23	0.97	0.26	0.08	0.20	0.16	0.04	1.03	0.81	0.22	0.08	1.12
Rong		21,585	203.85	4.40	70.40	97.42	97.42	0.00	0.29	0.29	0.00	0.06	0.06	0.06	0.00	0.23	0.23	0.00	0.06	0.28
Long		16,449	172.65	2.84	61.18	1843.80	1556.49	287.32	0.62	0.51	0.11	0.04	0.10	0.08	0.02	0.53	0.43	0.10	0.04	0.57
Duliu		11,326	202.19	2.29	356.54	380.98	380.98	0.00	0.08	0.08	0.00	0.14	0.04	0.04	0.00	0.05	0.05	0.00	0.14	0.18
Guyi		5098	256.96	1.31	100.20	511.78	511.78	0.00	0.08	0.08	0.00	0.02	0.01	0.01	0.00	0.07	0.07	0.00	0.02	0.09
Qian		198,005	196.56	38.92	12.85	1578.86	1365.27	213.60	8.16	6.86	1.30	0.08	1.43	1.20	0.23	6.73	5.66	1.07	0.08	6.81
Xun		308,271	198.23	61.11	57.54	1484.80	1323.09	161.71	11.86	10.32	1.53	0.65	2.31	2.02	0.30	9.54	8.31	1.24	0.65	10.20
Gui		19,288	263.89	5.09	124.59	697.73	697.73	0.00	0.36	0.36	0.00	0.10	0.05	0.05	0.00	0.31	0.31	0.00	0.10	0.41
He		11,536	246.19	2.84	492.02	1566.03	1299.54	266.49	0.28	0.23	0.05	0.23	0.07	0.06	0.01	0.21	0.18	0.03	0.23	0.44
Low Xi		353,100	186.83	65.97	113.65	1409.32	1222.38	186.94	11.06	9.34	1.72	1.56	1.66	1.39	0.27	9.40	7.95	1.45	1.56	10.96
